# Analysis of Drought Tolerance and Associated Traits in Upland Cotton at the Seedling Stage

**DOI:** 10.3390/ijms20163888

**Published:** 2019-08-09

**Authors:** Hai-Ming Li, Shao-Dong Liu, Chang-Wei Ge, Xiao-Meng Zhang, Si-Ping Zhang, Jing Chen, Qian Shen, Fei-Yan Ju, Yong-Fei Yang, Yang Li, Rui-Hua Liu, Hui-Juan Ma, Xin-Hua Zhao, Cun-Dong Li, Chao-You Pang

**Affiliations:** 1 State Key Laboratory of Cotton Biology, Institute of Cotton Research of CAAS, Anyang 455000, China; 2 Hebei Base of State Key Laboratory of Cotton Biology, Hebei Agricultural University, Baoding 071001, China; 3 Zhengzhou Research Base, State Key Laboratory of Cotton Biology, Zhengzhou University, Zhengzhou 450001, China

**Keywords:** association analysis, drought tolerance, upland cotton, single-nucleotide polymorphism (SNP)

## Abstract

(1) Background: Upland cotton (*Gossypium hirsutum* L.) is the most important natural fiber worldwide, and it is extensively planted and plentifully used in the textile industry. Major cotton planting regions are frequently affected by abiotic stress, especially drought stress. Drought resistance is a complex, quantitative trait. A genome-wide association study (GWAS) constitutes an efficient method for dissecting the genetic architecture of complex traits. In this study, the drought resistance of a population of 316 upland cotton accessions was studied via GWAS. (2) Methods: GWAS methodology was employed to identify relationships between molecular markers or candidate genes and phenotypes of interest. (3) Results: A total of 8, 3, and 6 SNPs were associated with the euphylla wilting score (EWS), cotyledon wilting score (CWS), and leaf temperature (LT), respectively, based on a general linear model and a factored spectrally transformed linear mixed model. For these traits, 7 QTLs were found, of which 2 each were located on chromosomes A05, A11, and D03, and of which 1 was located on chromosome A01. Importantly, in the candidate regions *WRKY70*, *GhCIPK6*, *SnRK2.6*, and *NET1A*, which are involved in the response to abscisic acid (ABA), the mitogen-activated protein kinase (MAPK) signaling pathway and the calcium transduction pathway were identified in upland cotton at the seedling stage under drought stress according to annotation information and linkage disequilibrium (LD) block analysis. Moreover, RNA sequencing analysis showed that *WRKY70*, *GhCIPK6*, *SnRK2.6*, and *NET1A* were induced by drought stress, and the expression of these genes was significantly different between normal and drought stress conditions. (4) Conclusions: The present study should provide some genomic resources for drought resistance in upland cotton. Moreover, the germplasm of the different phenotypes, the detected SNPs and, the potential candidate genes will be helpful for molecular marker-assisted breeding studies about increased drought resistance in upland cotton.

## 1. Introduction

Water shortages are among the most serious limitations to global agricultural production due to the complexity of water-deficient environments and climatic variation [1,2]. Drought is an important and complicated abiotic stress that affects the growth, development, and reproduction of plants. Drought limits cotton (*Gossypium hirsutum* L.) productivity and fiber quality, drought resistance is a complex, quantitative trait controlled by more than two genes [3,4]. It is, therefore, of great significance to improve the drought tolerance of cotton and increase its yield under drought stress conditions.

Allotetraploid upland cotton (*Gossypium hirsutum* L.; AADD, 2n = 4× = 52) is one of the most important economic crop species. It produces the most significant natural fiber worldwide, is a source of seed oil, and it accounts for approximately 95% of cotton production worldwide [5]. Upland cotton is the northern-most cultivated species and accounts for approximately 99% of the cotton planting area in China. Upland cotton has made important contributions to the development of modern cotton production and the cotton spinning industry [6,7]. Upland cotton is highly regarded by the government in relation not only to people’s lives and economic sources of cotton farmers but also to the economic development of cotton planting areas. As a glycophyte, cotton is more tolerant to abiotic stress than other staple crop species. However, during the past few years, the frequent occurrence of extreme weather events and environmental factors, such as drought stress, have affected the growth, development, yield, and fiber quality of cotton [8]. In Pakistan, cotton production decreased by 34% from 14.593 million bales in one year to 9.687 million bales the following year on account of water deficit [9]. China is one of the world’s largest producers and consumers of cotton [10]. With the rapid adjustment of cotton areas in China, cotton planters have moved to cities and towns and have begun participating in secondary and tertiary industries; thus, the major cotton planting region gradually has moved to the northwestern territories in China [11]. In recent years, the northwestern inland cotton region, which includes all of Xinjiang, the Hexi Corridor in Gansu, and the western region of the Helan Mountains of Inner Mongolia, has substantially developed and has become the largest cotton-growing region among three main cotton-growing regions (the cotton region in the Yangtze River basin, the cotton region in the Yellow River basin, and the northwestern inland cotton region) in China in terms of yield, total production, and planting acreage [12,13]. Water is one of the significant resources in arid and semiarid regions for farming. However, the northwestern inland cotton region is located just in the arid region in China, and the arid region in northwestern China is one of the most drought-stressed regions worldwide [14]. Therefore, it is a great challenge and formidable task for researchers to determine suitable drought resistance cultivation techniques and cultivate drought-tolerant cotton varieties.

Genome-wide association study (GWAS) methodologies are widely employed to identify relationships between molecular markers or candidate genes and phenotypes of interest. This method takes advantage of high-density SNP markers, associates molecular polymorphisms with a phenotype, and offers advantages over traditional linkage analyses such as more accurate positioning and mapping, simultaneous assessments of multiple alleles at a locus, and no requirements for linkage group construction [15,16]. Compared with biparental QTL studies, GWASs capture the available allelic diversity at loci while achieving greater physical resolution because of their lower linkage disequilibrium (LD) in diversity panels and because biparental mapping is unable to explore the full extent of allelic diversity that would be present in diverse germplasms [17]. Therefore, GWASs constitute an efficient method for dissecting the genetic architecture of complex traits and represent a powerful tool for crop breeding [18]. GWASs have been successfully applied in many plant species such as *Arabidopsis* [19], rice [20], maize [21], and wheat [22]. It has been successfully applied in cotton to elucidate the genetic basis of fiber quality [23,24], yield [25], verticillium wilt disease [26], and fusarium wilt disease [27]; however, this method has yet to be applied to drought resistance traits. Recently, with respect to cotton studies, the release of the genome sequence of Texas marker-1 (TM-1) has provided inestimable genomic information for future GWASs on other important traits in cotton [28,29]. Using significantly identified SNPs, GWAS analysis is a powerful tool for genomic selection and increases the accuracy of prediction. The general linear model (GLM) and mixed linear model (MLM) are the most commonly used algorithmic models in GWASs. In most previous related studies, these two models were often used in GWAS analyses. However, the computational complexity of the two models is tremendous. With the development of bioinformatics, efficient mixed model association expedited (EMMAX) has been used to study genotypes and phenotypic traits, it represents an efficient analytical method, and this method can reduce the computational time for analyzing large GWAS datasets from years to hours [30]. The factored spectrally transformed linear mixed model (FASTLMM) has also been used to analyze GWAS data and runs an order of magnitude faster than current efficient algorithms [31].

The use of genetic and genomic analyses to identify DNA markers associated with drought resistance can facilitate breeding strategies for crop improvement [17]. It is a useful and effective method to study target traits controlled by several genes, such as those that provide drought stress resistance. Screening materials in controlled greenhouse and growth chambers can greatly shorten the time needed and can improve the efficiency in screening for drought tolerance. Moreover, screening for upland cotton drought tolerance at the seedling stage in the greenhouse is conducive to the efficient screening of many materials in appropriate time and space. Based on previous sequencing data, the objectives of this study were to explore the genetic variation of 316 upland cotton accessions and use a GWAS to identify candidate regions and genes associated with drought resistance traits in order to develop tools for use in upland cotton breeding programs.

## 2. Results

### 2.1. Analysis of Phenotypic Drought Resistance Traits

**Figure 1:**
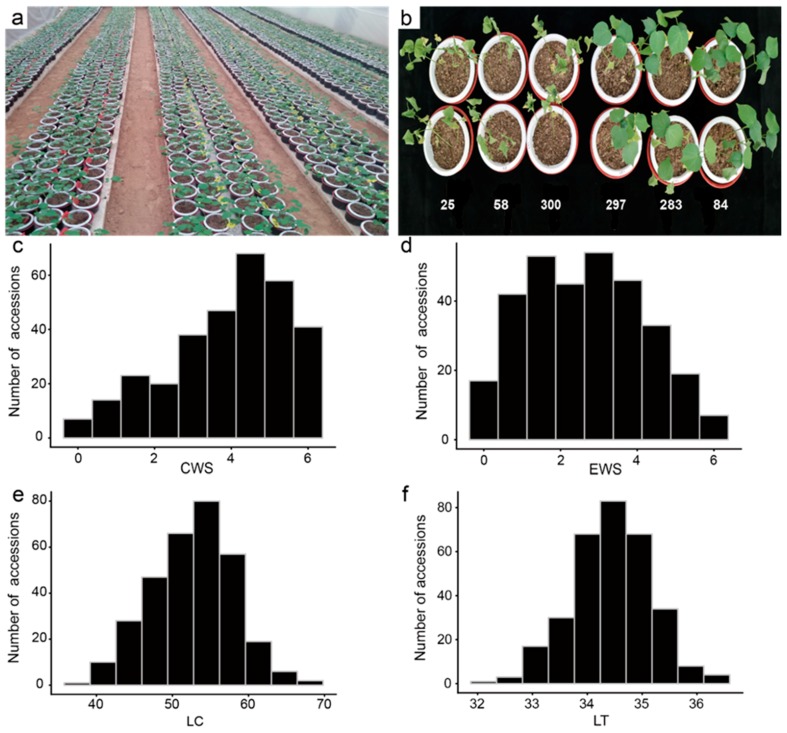
Phenotypes and frequency histograms of the mean values of drought resistance traits of 316 accessions. (**a**) Growth of the cotton population at the seedling stage under normal water conditions; (**b**) Typical phenotypes of the cotton population at the seedling stage after drought stress; (**c**–**f**) Frequency histograms of the mean values of the CWS, EWS, LC and LT traits of 316 accessions. CWS: cotyledon wilting score; EWS: euphylla wilting score; LC: leaf chlorophyll content; LT: leaf temperature.

In order to systematically explore the genetic basis and mechanism of drought resistance traits of upland cotton, a set of 316 accessions was selected from a natural population of 355 cultivars or lines (Figure 1a,b), and the leaf temperature (LT), cotyledon wilting score (CWS), euphylla wilting score (EWS), and leaf chlorophyll content (LC), which are target traits of these accessions related to drought resistance, were evaluated and analyzed under two different greenhouse environments (denoted as GR-2017 and GR-2018). For all 316 upland cotton accessions, the CWS and EWS ranged from 0 to 6 in both GR-2017 and GR-2018. The CWS had an average value of 4.0 and 3.9 in GR-2017 and GR-2018, respectively. The EWS had the same mean value of 2.5 in both environments, and the LC exhibited a wide range of 37.20–67.85 with a mean value of 52.70 in both environments (Figure 1c–f, Supplementary Table S1).

### 2.2. Phylogenetic Analyses and Linkage Disequilibrium

**Figure 2:**
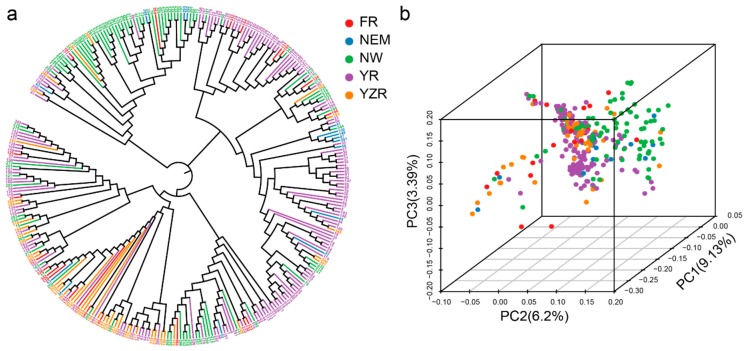
Population structure of 316 upland cotton accessions. (**a**) Phylogenetic tree of the natural population, FR: foreign region; NEM: the northern specific early maturation region; NW: the northwestern inland region; YR: the Yellow River basin region; YZR: the Yangtze River basin region; (**b**) Principal component analysis of upland cotton, each dot represents an accession.

To explore drought resistance of upland cotton, we collected a total of 316 upland cotton cultivars, including 286 cultivated accessions from the major cotton cultivation regions such as the Yangtze River basin region (YZR), the Yellow River basin region (YR), the northern specific early maturation region (NEM), and the northwestern inland region (NW) and foreign region (FR), including others from America, Tajikistan, and Uzbekistan. Based on the pair-wise genetic distance determined using whole genome SNP markers among the 316 accessions, we explored the phylogenetic relationships of these cotton germplasms, which could be divided into three divergent groups (Figure 2a). To further support the result, a principal component analysis (PCA) was performed by using select SNP markers. These groups could be identified according to principal components 1, 2, and 3 by a three-dimensional diagram (Figure 2b). Combining the results of the phylogenetic tree with population structure, we observed that most of these accessions from every subpopulation had mixed ancestry. We could not identify obvious geographic subpopulations, possibly because of the introduction of cotton to China from other countries in the early 20th century, which indicated that these accessions might have experienced gene flow or introgression in cotton breeding.

Linkage disequilibrium (LD) was indicated by the *r*^2^ value and decreased with physical distance between SNPs. By using all selected SNPs, the extent of LD for every group and all accessions was measured when the LD decreased to half of the maximum value. The observed extent of LD decay in the population was estimated to be 488 kb, with an *r*^2^ = 0.42 at half of the maximum value. According to the results of the LD analysis in conjunction with ecological region, the LD decay occurred at 351 kb (*r*^2^ = 0.42) in the YR region and at 408 kb (*r*^2^ = 0.42) in the NW region, but it increased to 584 kb (*r*^2^ = 0.43) in the YZR region (Figure S1).

### 2.3. Genome-Wide Association Study

There was abundant variation in the phenotype of cotton at the seedling stage under drought stress. To obtain insight into the genetic variants associated with drought tolerance at the seedling stage in upland cotton, based on the 316 cotton accessions, we investigated the phenotypic data for 4 traits and conducted a genome-wide association study to analyze and identify the genetic loci of those traits. For the analysis, multifarious distinct models, including the GLM and the FASTLMM, were used for different traits.

**Figure 3:**
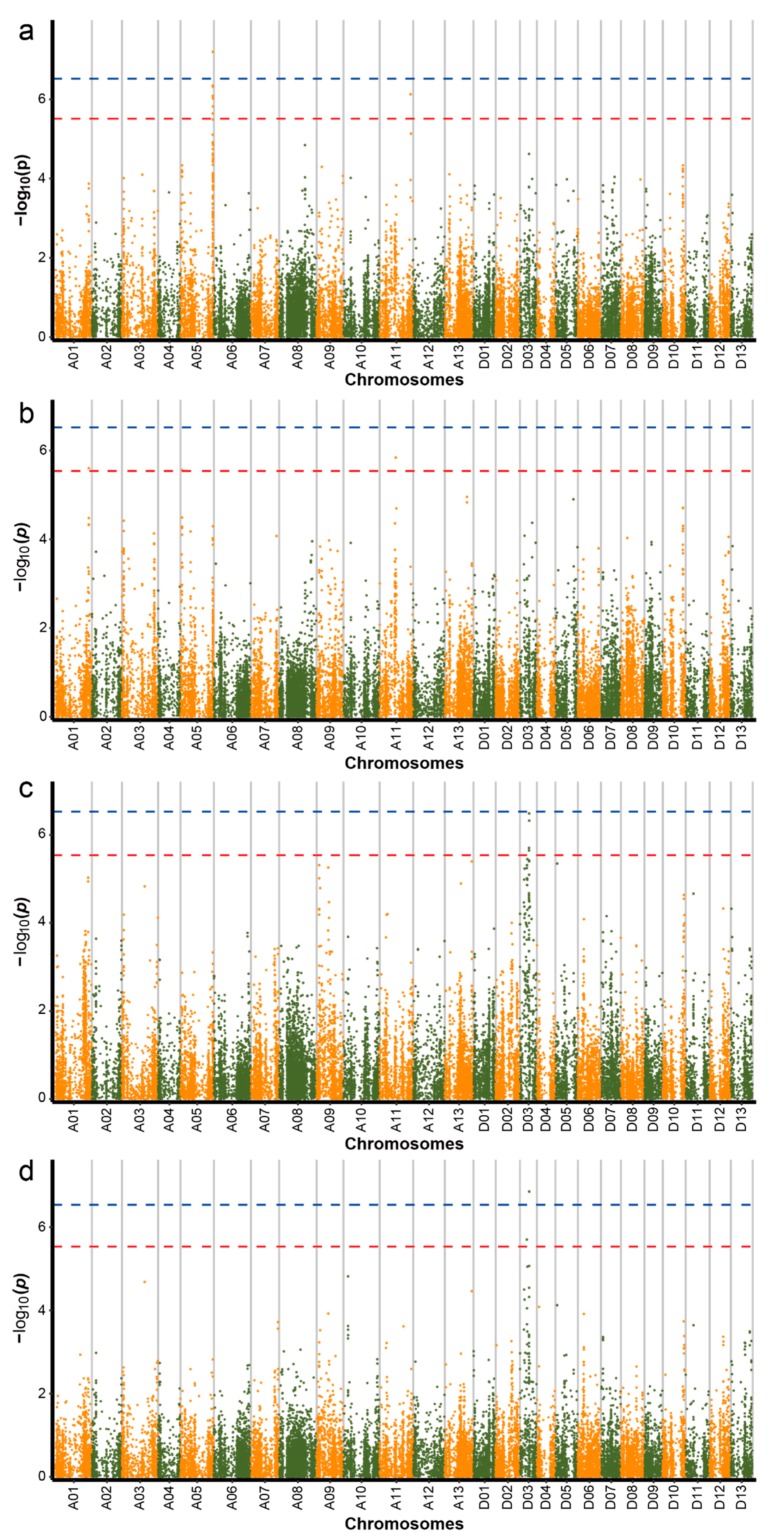
Genome-wide associated study (GWAS) of 316 upland cotton accessions. Manhattan plots of GWAS by using general linear model (GLM) for EWS (**a**), CWS (**b**), and LT (**c**), respectively and by using factored spectrally transformed linear mixed model (FASTLMM) for LT (**d**). The SNP loci of the red lines (−log10(*p*) ≥ 5.53) were considered suggestive association makers; the SNP loci of the blue lines (−log10(*p*) ≥ 6.53) were considered significant association makers. Each dot represents an SNP.

In this study, we used the GLM to compute the association signal of all four traits (no significant association makers were detected for the LC trait in this study) first. Seventeen SNP loci associated with three traits (LT, the EWS, and the CWS) related to drought tolerance and two SNP loci surpassed the threshold of significance of 0.01 (−log10(*p*) ≥ 6.53) (Table 1 and Figure 3). With respect to these SNP loci, 64.7% and 35.3% were situated in the A subgenome and D subgenome, respectively; 47.1% were distributed on chromosome A05; and 11.8% and 35.2% were distributed on chromosome A11 and chromosome D03, respectively. Among these associated SNP loci, eight associations with the EWS were identified (Figure 3a), three associations with the CWS were identified (Figure 3b), and six associations with LT were identified (Figure 3c). In the FASTLMM, 3 associations were found to be significant for three SNP loci associated with LT (Figure 3d). However, no peak SNPs from this model were detected for the CWS and EWS (Supplementary Table S2).

### 2.4. Prediction of Candidate Genes and Identification of Favorable Haplotypes

**Figure 4:**
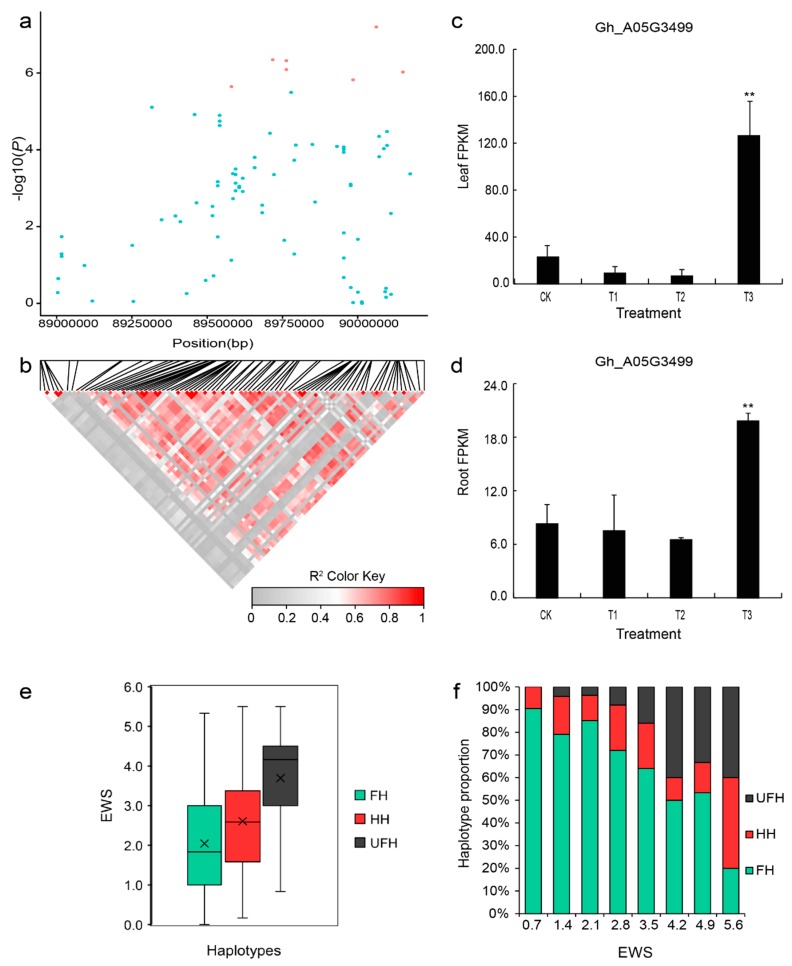
LD block on chromosome A05 and identification of the candidate gene *Gh_A05G3499*. (**a**) Association signal of the EWS in the region from 89.0–90.6 Mb on chromosome A05. (**b**) Distribution of the LD block on chromosome A05. The pair-wise LDs between the SNPs are indicated as D’ values, where the grey and dark red colors indicate 0 and 1, respectively. (**c**,**d**) Expression level of the candidate gene *Gh_A05G3499* (*WRKY70*) calculated based on RNA-seq analysis of leaves and roots. (**e**) Box plots for phenotypic values of the haplotypes on chromosome A05 for the EWS trait. (**f**) Percent stacking bar chart of several types of haplotypes for the EWS trait. CK: 75% ± 5% of RWC; T1: 60% ± 5% of RWC; T2: 45% ± 5% of RWC; T3: 30% ± 5% of RWC. The asterisks indicate the significance of Student’s t-test and ** means 1% level of significance. FH: favorable haplotype; HH: heterozygous haplotype; UFH: unfavorable haplotype; EWS: euphylla wilting score. The black multiplication sign indicates the mean value, the middle black line indicates the median, the box represents the range from the 25th to 75th percentile of all the data, and the outer dots are outliers.

To further explore drought tolerance based on EWS traits, we analyzed the candidate genes associated with these traits in depth. In the genomic region from 89.0–90.6 Mb, seven SNPs were found, and the SNP locus A05:90061632 showed significant marker–trait associations, with −log10(*p*) values as high as 7.14 (Figure 2a). A significant association was found to peak at SNP A05:89717711. However, the flanking sequence of the SNP (T/C) aligns with the intronic region of the *Gh_A05G3447* gene, which is annotated as an unknown protein. Moreover, we found two significant loci at SNP A05:89762730 and SNP A05:89762936, which are located at 89.76 Mb on chromosome A05. Unfortunately, although two SNPs (C/G, G/A) were located within the same *Gh_A05G3452* gene that encodes nuclear RNA polymerase C2, their flanking sequences were in the position of the intronic region, making it highly difficult for us to further study them. We further performed an LD block analysis for six significant SNPs (A05:89717711, A05:89762730, A05:89762936, A05:89984532, A05:90061632, and A05:90150044) associated with the EWS trait. One LD block (89.70–90.60 Mb) was ultimately found on chromosome A05 (Figure 4a,b). According to the result of the gene annotation, there were 61 putative genes in this LD block (Supplementary Table S3). To analyze the genes in this LD block, we further performed a transcriptome sequencing analysis for the accession (G8). We identified a gene, *Gh_A05G3499*, which was named *WRKY70*. With increasing drought stress, the expression of this gene first declined but then significantly increased in the leaves and roots (Figure 4c,d).

We also analyzed the types of haplotypes for the EWS trait to further find favorable haplotypes for the six significantly associated SNPs. According to results of the association analysis, three kinds of haplotypes (TT-CC-GG-AA-GG-AA, TC-CG-GA-AG-GA-AG, and CC-GG-AA-GG-AA-GG) were found on the basis of these SNP alleles (T/C, C/G, G/A, A/G, G/A, and A/G). The haplotype (TT-CC-GG-AA-GG-AA) included 109 accessions, accounted for 71.7% of all the haplotypes, and was used as the favorable haplotype (FH) because this haplotype had positive phenotypic effects on the EWS and showed less leaf wilting than the other haplotypes. The mean value (2.0) of the favorable haplotype (FH) for the EWS trait was obviously lower than the average (3.7) of the corresponding haplotype (CC-GG-AA-GG-AA-GG, considered unfavorable haplotype, UFH), which included 19 accessions. The third haplotype was considered a heterozygous haplotype (HH, TC-CG-GA-AG-GA-AG) and included 24 accessions, with the mean value of 2.9 for EWS trait (Figure 4e). According to the result of the percent stacking bar chart, the favorable haplotype (FH) accounted for a greater proportion of the lines with less leaf wilting than the other haplotypes. When the EWS was equal to or less than 0.7, there were no unfavorable haplotype (UFH) accessions, and favorable haplotype (FH) accounted for 90.5%. However, while the value was equal to or greater than 4.9, the favorable haplotype (FH) accounted for only 20%, and the unfavorable haplotype (UFH) and heterozygous haplotype (HH) accounted for 40% each (Figure 4f).

**Figure 5:**
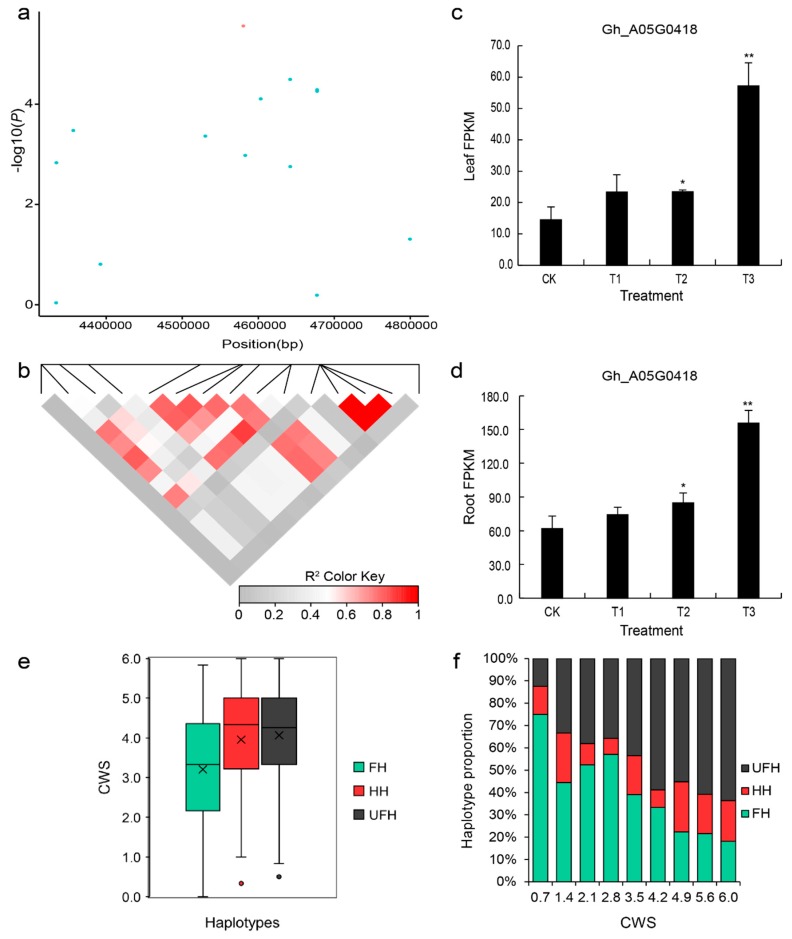
LD block on chromosome A05 and identification of the candidate gene *Gh_A05G0418*. (**a**) Association signal of the CWS in the region from 4.30–4.83 Mb on chromosome A05. (**b**) Distribution of the LD block on chromosome A05. The pair-wise LDs between the SNPs are indicated as D’ values, where the grey and dark red colors indicate 0 and 1, respectively. (**c**,**d**) Expression level of the candidate gene *Gh_A05G0418* (*CIPK6*) calculated based on RNA-seq analysis of the leaves and roots. (**e**) Box plots of the phenotypic values of the haplotypes on chromosome A05 for the CWS trait. (**f**) Percent stacking bar chart of several types of haplotypes for the CWS trait. CK: 75% ± 5% of RWC; T1: 60% ± 5% of RWC; T2: 45% ± 5% of RWC; T3: 30% ± 5% of RWC. The asterisks indicate the significance of Student’s t-test, and * and ** mean the 5% and 1% levels of significance, respectively. FH: favorable haplotype; HH: heterozygous haplotype; UFH: unfavorable haplotype; CWS: cotyledon wilting score. The black multiplication sign indicates the mean value, the middle black line indicates the median, the box represents the range from the 25th to 75th percentile of all the data, and the outer dots are outliers.

When the seedlings of upland cotton were under drought stress, we found that the cotyledons of seedlings wilted early, after which the euphylla became gradually wilted. The degree of wilting was asynchronous in numerous upland cotton accessions with different drought resistances. Here, the underlying genetic loci associated with the CWS (cotyledon wilt score) trait were also detected. Three SNPs were significantly associated with the CWS trait; these SNP loci were positioned at 9,221,3571, 4,580,354, and 45,127,798 on chromosomes A01 (1), A05 (1), and A11 (1), respectively (Figure 3b). We analyzed a peak SNP, which was located at 4,580,354 on chromosome A05. This SNP had a MAF of 0.42 in the natural population and led to delimiting a 0.45 Mb candidate region that included 46 genes (Supplementary Table S4). Moreover, one LD block (4.30–4.83 Mb) was found on chromosome A05 (Figure 5a,b). When analyses of the candidate region and LD block were combined, one gene, *Gh_A05G0418*, was found to be located approximately 0.14 Mb away from the peak SNP A05:4580354 and encoded CBL-interacting protein kinase 6 (CIPK6), which is homologous to the gene *AT4G30960* in *Arabidopsis*. Based on the results of RNA-seq, with increasing drought stress, the expression of this gene gradually increased, after which it ultimately peaked and reached an extremely significant expression in leaves and roots of the accession compared to control treatments under severe drought conditions (Figure 5c,d). Moreover, we found that the gene showed a significant expression level under the same treatment (T3) in the leaves and roots of the variety.

We further analyzed the types of haplotypes for the CWS trait to find favorable haplotype for significantly associated SNPs. With respect to the wilting score mean of the three types of haplotypes, the favorable haplotype (AA), heterozygous haplotype (AT), and unfavorable haplotype (TT) scores were 3.21, 3.95, and 4.07, respectively. In the natural population, the favorable haplotype (FH) accounted for 50%, the heterozygous haplotype (HH) accounted for 34%, and the unfavorable haplotype (UFH) accounted for only 16% (Figure 5e). Based on the result of the percent stacking bar chart, different wilting scores were distributed among the three types of haplotypes. As can be seen, as the wilting score increased gradually, the proportion of the favorable haplotype (FH) decreased (Figure 5f).

**Figure 6:**
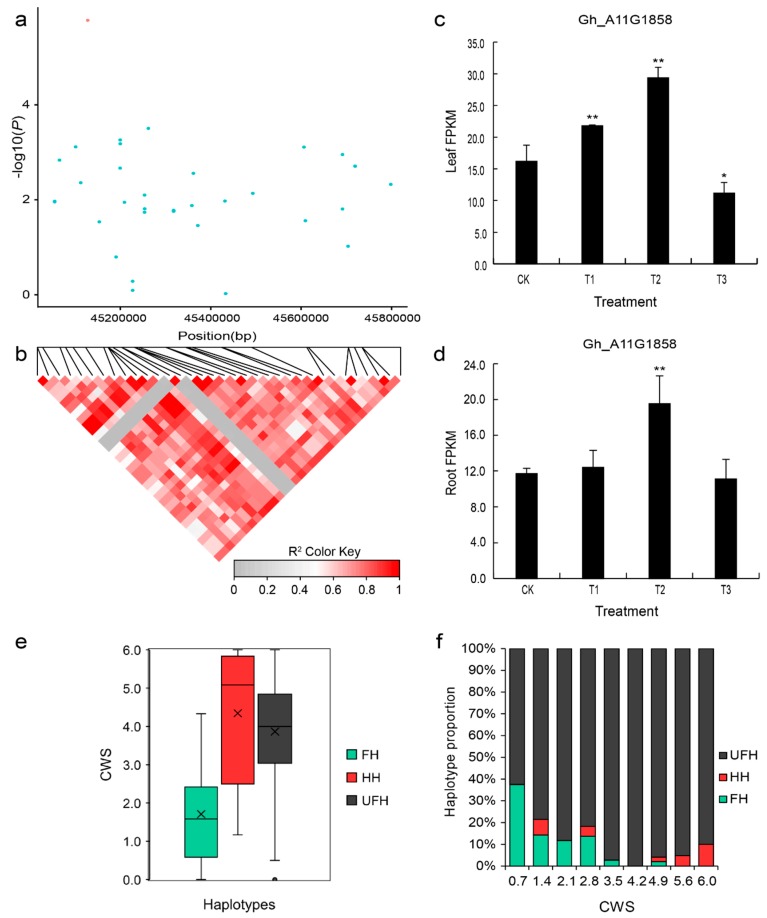
LD block on chromosome A11 and identification of the candidate gene *Gh_A11G1858*. (**a**) Association signal of the CWS in the region from 45.0–45.8 Mb on chromosome A11. (**b**) Distribution of the LD block on chromosome A11. The pair-wise LDs between the SNPs are indicated as D’ values, where the grey and dark red colors indicate 0 and 1, respectively. (**c**,**d**) Expression level of the candidate gene *Gh_A11G1858* (*SnRK2.6*) calculated based on RNA-seq analysis of the leaves and roots. (**e**) Box plots of the phenotypic values of the haplotypes on chromosome A11 for the CWS trait. (**f**) Percent stacking bar chart of several types of haplotypes for the CWS trait. CK: 75% ± 5% of RWC; T1: 60% ± 5% of RWC; T2: 45% ± 5% of RWC; T3: 30% ± 5% of RWC. The asterisks indicate the significance of Student’s t-test, and * and ** mean the 5% and 1% levels of significance, respectively. FH: favorable haplotype; HH: heterozygous haplotype; UFH: unfavorable haplotype; CWS: cotyledon wilting score. The black multiplication sign indicates the mean value, the middle black line indicates the median, the box represents the range from the 25th to 75th percentile of all the data, and the outer dots are outliers.

In addition, we further analyzed another peak SNP associated with the CWS trait, which was at position 45,127,798 on chromosome A11 and had a MAF of 0.06 in the population; the R2 value for this association was 9.64%. The marker explained 9.64% of the variation. Moreover, one LD block (45.0–45.8 Mb) was found on chromosome A11 (Figure 6a,b). There were three predicted genes in the designated candidate region (Supplementary Table S4). One gene, *Gh_A11G1858*, was homologous to the *AT4G33950* gene in *Arabidopsis thaliana*. The gene *AT4G33950* is named *SnRK2.6*/*OST1* (sucrose non-fermenting 1-related protein kinase 2.6/OPEN STOMATA 1), which encodes a protein kinase superfamily protein. According to the RNA-seq analysis, the expression of this gene was gradually upregulated under mild (T1) and moderate drought conditions (T2) but decreased under the severe drought conditions in the leaves and roots of the accession (Figure 6c,d). Moreover, the expression of this gene was significant in T2 compared to control treatments in the leaves.

Based on the analysis of the haplotypes, the average values of the wilting score of the three haplotypes, favorable haplotype (CC), heterozygous haplotype (TC), and unfavorable haplotype (TT) were 1.71, 4.34, and 3.87, respectively (Figure 6e). Based on the results of the percent stacking bar chart, the favorable haplotype (FH) accounted for only 5% of the natural population and was distributed in regions associated with a relatively low wilting score (Figure 6f).

**Figure 7:**
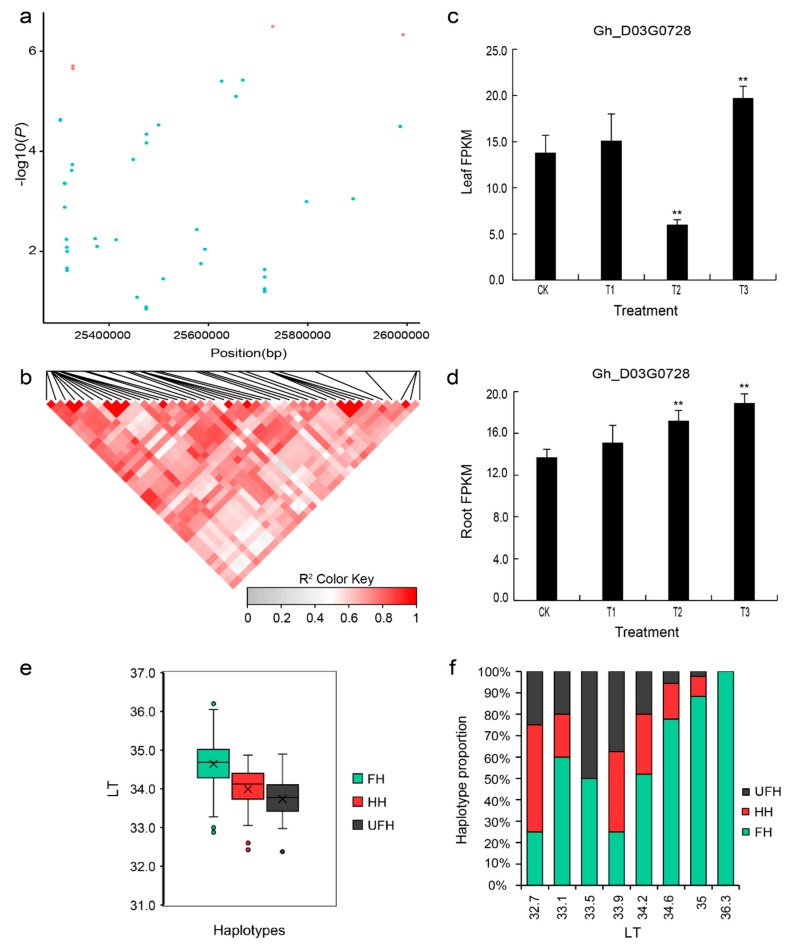
LD block on chromosome D03 and identification of the candidate gene *Gh_D03G0728*. (**a**) Association signal of the LT in the region from 25.3.0–26.0 Mb on chromosome D03. (**b**) Distribution of the LD block on chromosome D03. The pair-wise LDs between the SNPs are indicated as D’ values, where the grey and dark red colors indicate 0 and 1, respectively. (**c**,**d**) Expression level of the candidate gene *Gh_D03G0728* calculated based on RNA-seq analysis of the leaves and roots. (**e**) Box plots of the phenotypic values of the haplotypes on chromosome D03 for the LT trait. (**f**) Percent stacking bar chart of several types of haplotypes for the LT trait. CK: 75% ± 5% of RWC; T1: 60% ± 5% of RWC; T2: 45% ± 5% of RWC; T3: 30% ± 5% of RWC. The asterisks indicate the significance of Student’s t-test and ** means and 1% levels of significance. FH: favorable haplotype; HH: heterozygous haplotype; UFH: unfavorable haplotype; LT: leaf temperature. The black multiplication sign indicates the mean value, the middle black line indicates the median, the box represents the range from the 25th to 75th percentile of all the data, and outer dots are outliers.

To further explore drought tolerance based on the LT trait, we analyzed the candidate genes associated with these traits in depth. In the genomic region from 25.3–26.0 Mb, five SNPs (D03:25327187, D03:25327414, D03:25669014, D03:25729410, and D03:25991794) were found (Figure 3c,d). When analyses of the candidate region and LD block were combined (Figure 7a,b), 48 genes were found in the candidate region (Supplementary Table S5). The gene *Gh_D03G0728* is named *NET1A*, which is homologous to *AT4G30960* (*CIP1*) in *Arabidopsis* and encodes COP1-interactive protein 1. This gene was induced in the leaves and roots in response to drought stress (Figure 7c,d).

Additionally, we analyzed the types of haplotypes for the LT trait to further identify favorable haplotype for five significantly associated SNPs. Similarly, there were three types of haplotype (AA-TT-AA-AA-AA, AT-TC-AC-GA-AG, and TT-CC-CC-GG-GG) based on the SNP alleles (A/T, T/C, A/C, G/A, and A/G). Because osmotic adjustment substances such as abscisic acid (ABA) accumulate under drought stress, osmotic pressure in guard cells gradually increases and induces stomatal pore apertures to become narrow or to completely close. As a result, plant transpiration decreases, and the temperature of leaves consequently increases. This mechanism is more important for drought-resistant plants in order to protect their growth and development process than for drought-sensitive materials. Therefore, the haplotype AA-TT-AA-AA-AA was regarded as the favorable haplotype (FH) because the mean value (34.6 ℃) of the haplotype for the LT trait was higher than the average (33.7 ℃) of the corresponding haplotype (TT-CC-CC-GG-GG, considered an unfavorable haplotype (UFH)). The mean (34.0 ℃) of the haplotype AT-TC-AC-GA-AG was in between that of the favorable haplotype (FH) and the unfavorable haplotype (UFH); this haplotype was regarded as a heterozygous haplotype (HH) (Figure 7e). Although the LT was equal to or greater than 35.4 ℃, the unfavorable haplotype (UFH) and heterozygous haplotype (HH) were not found among the natural population (Figure 7f).

## 3. Discussion

Drought stress significantly affects the growth and development, yield, and fiber quality of cotton by restricting and affecting plant height, leaf weight, stem weight, node number, transpiration rate, stomatal conductance, and so on. [8]. The seedling stage is one of the critical stages in the upland cotton growth cycle and especially lays the central foundation for subsequent growth. Although the seedling stage of upland cotton frequently encounters drought stress, there have been few studies of drought stress in upland cotton via genome-wide association studies thus far. Recently, the drought resistance of cotton treated with polyethylene glycol 6000 (PEG 6000) was reported [32]. Many genome-wide association studies have been employed to dissect complex traits in plants under drought stress and have proven to be effective for identifying candidate regions and candidate genes for these traits. In this study, a genome-wide association study was performed for phenotypic traits of cotton growing in soil under drought stress conditions at the seedling stage. The responses of 316 upland cotton accessions from different ecological regions to drought stress greatly differed according to phenotype traits (CWS, EWS, LT, and LC). In the present study, LD was calculated by using 33,557 SNPs averaged approximately 400 kb across the whole genome. LD decay was higher when SLAF sequencing technologies were used rather than resequencing or next-generation sequencing technologies, which find and identify high densities of SNPs and have been used in recent studies [23]. The higher LD in this study means fewer SNP loci were found to cover the whole genome. According to our analysis of the phylogenetic relationship of the natural population, we found that Stoneville 2B (STV2B) was divided into a YR (Yellow River basin region) group, in which some accessions were together with Deltapine 20, and a YZR group, which included Deltapine 14, Deltapine 15, and Deltapine 531. The results of this study are similar to those in that modern Chinese cultivars developed from Stoneville 2B (STV2B) were determined to be planted mainly in the Yellow River basin cotton-growing region, and Deltapine were determined to be planted in all major cotton-growing regions in China [25].

According to the results of the GWAS of the 316 upland cotton accessions, 17 SNPs were found on chromosomes A01, A05, A11, and D03 via other traits (the CWS, the EWS, and LT). Plant cells are subjected to a series of physiological reactions under drought or osmosis stress; water extrusion, reduction in osmotic potential (OP), and even plasmolysis can occur in the mesophyll cells of plants, which results in leaf rolling and ultimately gives rise to wilting of the plant leaves with increasing drought stress [33,34]. Leaf wilting of the upland cotton accessions from different ecological areas was not synchronized, and the leaves started to wilt after the cotyledon seedlings wilted. Moreover, the phenotypic data of the CWS were different from the EWS, which was verified by results from the association analysis. Generally, on account of the existence of LD, the deviation, and lack of data collection and statistical analysis, some of the most significant SNPs may not lie within the authentic loci in terms of reinforcing the significance to verify the SNPs [35]. LD may be combined with the use of SNPs in marker-assisted selection (MAS) if the linkage is strong enough to restrain the recombination that was found between the SNP and causal gene, which can emerge as governing the favorable phenotype. Moreover, methods used in molecular breeding programs for reducing SNPs have been explored in sugar beets [36].

One QTL that contained 61 and 14 genes was found on chromosomes A05 and A11, respectively, for the EWS trait (Supplementary Tables S3 and S6). According to annotation information and RNA-seq data, the gene *Gh_A05G3499* is homologous to *AT3G56400* in *Arabidopsis*, which encodes WRKY DNA-binding protein 70 (WRKY70). WRKY70, with one WRKY domain and a CCHC zinc finger motif, is a member of the WRKY family [37], and many studies have indicated that WRKY TFs play a significant role in the abiotic stress response [38,39]. In addition to their critical role in the plant abiotic stress response, WRKY proteins have been implicated in the regulation of developmental processes such as leaf senescence and trichome development [37]. Furthermore, studies have found that WRKY54 and WRKY70 cooperate as negative regulators of osmotic stress and that WRKY46, WRKY54, and WRKY70 negatively regulate drought resistance in *Arabidopsis* [40,41]. In this study, the gene *Gh_A05G3499* (*WRKY70*) was induced in the leaves and roots by drought stress. According to the results of RNA sequencing, we inferred that this gene not only may be involved in drought stress but also may regulate developmental processes. In the candidate region of SNP A05:4580354 for the CWS trait, we found 46 genes according to the LD block, six of which had an unknown function, and other annotated genes. 

The gene *Gh_A05G0418* is homologous to *AT4G30960*, which encodes CBL-interacting protein kinase 6 (CIPK6) in *Arabidopsis thaliana*. Previous studies revealed that the transcripts of the *AtCIPK6* gene largely accumulated in abscisic acid-treated seedlings compared to basal level expression, and overexpression of the *AtCIPK6* gene enhanced plant resistance to salt stress but increased sensitivity to ABA [42]. In *Brassica napus*, researchers found that activation of BnCIPK6 confers ABA hypersensitivity to *Arabidopsis* plants, and overexpression of *BnCIPK6* in *Arabidopsis cipk6* mutants completely rescued the ABA-insensitive phenotypes of this mutant, which further indicates that *BnCIPK6* is involved in the plant response to ABA [43]. Similarly, expression of the *GhCIPK6* gene was induced by salt, drought, and ABA treatments in *Gossypium hirsutum*. Moreover, overexpression of *GhCIPK6* significantly increases tolerance to salt, drought, and ABA stresses in transgenic *Arabidopsis thaliana*, which suggests that *GhCIPK6* plays a significant role and can serve as a positive regulator in response to salt and drought stresses [44]. Another gene, *Gh_A11G1858*, on chromosome A11 is named *SnRK2.6*/*OST1* and encodes a protein kinase superfamily protein. The sucrose non-fermenting-1-related protein kinase 2 (SnRK2) family consists of ten members in both the *Arabidopsis* (SnRK2.1–10) and rice (known as osmotic stress/ABA-activated protein kinases (SAPK1–10)) genomes, and the members are serine/threonine protein kinases that function as central and positive regulators of the ABA signaling pathway [45]. Half of SnRK2s are activated by ABA, including SnRK2.2, 2.3, 2.6, 2.7, and 2.8 [46]. These proteins are key regulators of the ABA signaling pathway and play a significant role in controlling stomatal regulation in response to drought stress [47,48]. 

Moreover, according to KEGG pathway and mapping analyses, the *SnRK2.6* gene plays a role in the mitogen-activated protein kinase (MAPK) signaling pathway and is induced by salt, drought, and osmotic stresses. MAPK signaling genes activate various drought-related pathways to induce tolerance in plants [49]. Leaf temperature (LT) is a measurement associated with multiple abiotic stresses and is a drought tolerance-related QTL trait [50]. Previous research found one QTL on chromosome A06 [51] and on chromosome D03 for the LT trait [4]. Similarly, all SNPs we found were located on chromosome D03. In the candidate region for the LT trait, the gene *Gh_D03G0728* (*NET1A*) was induced by drought stress in this study. With respect to the homologous gene (*CIP1*), a previous study indicated that the promoter activity of *CIP1* can be induced by osmotic stress and ABA, and that cip1 loss-of-function mutants have ABA-insensitive phenotypes [52]. We infer that this gene may be involved in drought resistance. QTLs controlling chlorophyll content were studied and identified on chromosomes A02, D05, and D10 via QTL analyses for cotton near-isogenic lines under drought stress [53], and these QTLs were found on chromosomes A02 and D01 via the Cotton Marker Database based on G. *hirsutum* × G. *barbadense* [51]. However, in this study, the leaf chlorophyll content (LC) was not significantly associated with any loci in plants under drought stress.

In addition, in recent years, genes involved in drought resistance have been gradually found in upland cotton; some of these genes encode transcription factors, such as *GhMYB5* and *GhMAP3K40* [54,55], and other genes are involved in calcium-sensing, such as *GhCIPK6*, *GhCDPK2*, and *GhCDPK38* [44]. According to the latest study of drought stress in cotton, the *RD2*, *HAT22*, and *PIP2* genes were associated with drought tolerance and were identified via water culture experiments of cotton plants treated with PEG 6000 [32]. Most of these genes have been studied for their response to drought stress, which was still in the early stages or was performed in chemical reagent environments. Moreover, it is extremely difficult for transgenic drought-resistant cotton to develop and even advance to field production at present, as drought tolerance is a complex, quantitative trait that is controlled by multiple genes. Hence, the study of drought tolerance in upland cotton still needs to be further explored, especially in terms of the molecular mechanism of drought tolerance. Indeed, in order to study the mechanism of gene or genes directly involved in the phenotype, it would be indispensable to knock out the genes in these regions by methods such as those involving RNA interference, T-DNA, or CRISPR/Cas9 and observe the resultant phenotype [56].

## 4. Materials and Methods 

### 4.1. Plant Materials

The diversity panel for the GWAS consisting of 316 upland cotton (*Gossypium hirsutum* L.) accessions was provided by the cotton early maturing breeding laboratory at the Cotton Research Institute of Chinese Academy of Agricultural Sciences (CRI-CAAS). The natural population comprising 316 upland cotton cultivars or lines (Supplementary Table S1) used for the association analysis in this study were from China, America, Uzbekistan, Israel, Tajikistan, and Azerbaijan, which aimed to capture a high geographical diversity. The cultivars or accessions were divided into five groups according to ecological area: (1) the NEM group (15 accessions from the northern specific early maturation region in China), (2) the NW group (87 accessions from the northwestern inland region of China), (3) the YR group (151 accessions from the Yellow River valley region in China), (4) the YZR group (42 accessions from the Yangtze River valley region in China), and (5) the FR group (21 accessions from the United States of America and 2 accessions from west and central Asia countries). The 316-genotype subset of the natural population consisting of 355 cultivars or accessions in terms of cotton lint percentage, fiber quality, and early maturity was selected on the basis of existing data and previous knowledge of genome sequencing data under nonstress conditions [57]. All accessions had been self-pollinated for more than four generations by planting under natural growing conditions.

### 4.2. Locations and Phenotyping

All 316 upland cotton cultivars and lines were planted during the winter and spring of 2017 and 2018 (designated GR-2017 and GR-2018, respectively) in the greenhouse at the Cotton Research Institute of the Chinese Academy of Agricultural Sciences (CRI-CAAS), Anyang, Henan, China (36°08′ N, 114°48′ E). Summer seasons were avoided to limit confounding factors from extremely high heat and the abundance of mixed gas and airflow produced by the fan drafts and cooling systems in the greenhouse. The greenhouse experimental design consisted of randomized complete blocks with three replications; each replication per material consisted of two 2000 cm^3^ plastic pots. A soil mixture of (3:1:0.5 topsoil, substrate, and vermiculite, respectively) with 40 g of carbendazim (50%) per cubic meter of mixture, was uniformly mixed and covered with plastic paper to sterilize the germplasm, which were then exposed to air after 48 h. The seeds of the germplasm were soaked in water for 24 h with carbendazim (500 times 50%) before planting. All pots were filled with 450 g of the soil mixture and were watered to field capacity; excess water was allowed to drain for 48 h before planting. Each genotype was planted with six seeds that were visually selected for having similar size and good quality in each pot. Each pot was watered with 100 mL with a graduated cylinder every four days after cotton emergence and then watered with 200 mL with a graduated cylinder at one-leaf stage every four days. All seedlings were thinned to three plants per pot at one-leaf stage approximately 15 d post-planting, and watering was stopped when the plants were at the three-leaf stage. The leaf chlorophyll content (LC) of individual plants was measured by using a leaf chlorophyll meter (LC-502, Soil-Plant Analysis Development (LC) Section, Minolta Camera Co., Osaka, Japan), and the leaf temperature of the second leaf from the top of each plant was measured. In addition, the volumetric water content (VWC) of the soil in each pot was measured daily for 35 d after planting, and individual plants were scored for wilting. The wilting was scored every day for 7 consecutive days; the wilting was scored on a scale of 0 to 6, with 0 being no sign of wilting and 6 being completely wilted [58].

### 4.3. Genotyping and Phylogenetic Analysis

The 355 cotton accessions or varieties were genotyped using SLAF-seq with an Illumina HiSeq 2500 system (Illumina, Inc., San Diego, CA, USA) at Biomarker Technologies Corporation in Beijing. A sequencing dataset consisting of 355 cotton accessions or varieties was generated and consisted of 96.10 Gb, which included 874.44 million paired-end reads with a length of ~80 bp. According to the minor allele frequency (MAF), 81,675 SNPs with a MAF ≥ 0.05 were ultimately used for analyses, and the 81,675 SNP markers covered all 26 chromosomes (http://www.ncbi.nlm.nih.gov/bioproject/PRJNA314284/SRP071133) [59]. Based on these sequencing datasets, the genomic data were selected again and mapped against the TM-1 genome to identify genomic variants [29]. According to the standards of filtrating (integrity ≥ 0.5 and MAF ≥ 0.05), 33,557 SNPs were filtered from the sequencing dataset of the 316 upland cotton cultivars or lines. These selected SNPs were used, and a phylogenetic tree of all 316 accessions was constructed by MEGA 5 [60] software together with the neighbor-joining algorithm (1000 bootstraps).

### 4.4. Population Structure and Linkage Disequilibrium (LD) Decay Analysis

The population structure of all 316 accessions was estimated via Admixture software for individual ancestry and allele frequency correlations. Ten thousand iterations were performed with the maximum likelihood method, and the number of populations (k) was tested from 2 to 20. The relative kinship between individuals was calculated by SPAGeDi [61] software. Based on all the SNPs, the structure of the cotton population of the 316 cultivars or lines was analyzed by the principal component analysis (PCA) method of EIGEN [62] software. Pair-wise LD between markers was estimated as the squared correlation coefficient (*r*^2^) of alleles by using GAPIT [63] software.

### 4.5. Genome-Wide Association Study Analysis

For all the SNPs from the 316 accessions and phenotypic data, we used the GLM with Q and K matrices to perform an association analysis between the wilting, LC, and genetic data. Kinship was derived from all the SNPs. The GLM was applied by using TASSEL version 5.2.34 software, and FASTLMM was applied by using FASTLMM software for the analysis of genome-wide associations [31]. Bonferroni-corrected *p* values of ≤0.1 (*p* = 0.1/*n* = 2.98 × 10^−6^; *n* = total markers used, −log10(*p*) = 5.53, red line in the Manhattan plots) and 0.01 (*p* = 0.01/*n* = 2.98 × 10^−7^ *n* = total markers used, −log10(*p*) = 6.53, blue line in the Manhattan plots) were used as a threshold of significance and a suggestive threshold to estimate whether significant associations existed for the GWAS. Based on the GWAS results, SNP loci significantly associated with drought resistance traits were further analyzed.

### 4.6. Haplotype Analysis

Each haplotype was selected by the types of SNPs associated with the target traits. The corresponding phenotypic value was calculated based on the average of the phenotypic value among all accessions in the haplotype. According to the research objective of each target trait, the favorable haplotype (FH) and other haplotypes were analyzed by using R software. On the basis of the corresponding phenotypic value of the different haplotypes, box plots of each haplotype were generated by using R software, and then percent stacking bar charts of several types of haplotypes were analyzed by using statistical software.

### 4.7. RNA-seq Analysis

For the transcriptome analysis, we selected upland cotton accession G8, which was screened and identified in a growth chamber at CRI-CAAS. Eight upland cotton leaf samples and root samples, including three biological replicates of a single plant per replicate, were used for RNA sequencing. Leaves and roots at four treatment times—the CK (RWC 75% ± 5%), T1 (RWC 60% ± 5%), T2 (RWC 45% ± 5%), and T3 (RWC 30% ± 5%)—were harvested, immediately frozen in liquid nitrogen, and then stored at −80 ℃, after which they were prepared for RNA extraction. For each sample, total RNA was isolated using Column Plant RNAout (Tiandz, Beijing, China). The quality and concentration of RNA were then verified using a NanoDrop^TM^ 2000 UV-vis spectrophotometer (Thermo Scientific, Waltham, MA, USA). An Agilent 2100 Bio-analyzer (Agilent Technologies, Santa Clara, CA, USA) and RNase-free agarose gel electrophoresis were also used to qualify and quantify the 48 samples. The mRNA was subsequently enriched using oligo (dT) magnetic beads (Qiagen) and then broken into short fragments. The Illumina HiSeq™ 2000 platform using paired-end (PE) technology with raw reads of approximately 100 bp in length of Gene Denovo Co (Guangzhou, China) was used to generate transcriptome sequences. Clean reads were selected by removing low-quality reads, adaptor-containing reads, and reads containing >5% N bases. A false discovery rate (FDR) was applied to determine the threshold of *p*-values in multiple tests and analyses. Moreover, the DEGs were obtained according to FDR ≤ 0.005 and |log2FC| ≥ 1.5.

## Figures and Tables

**Table 1:** Summary of loci associated with drought tolerance traits identified by a GWAS in upland cotton.

Trait	SNP Locus	Chromosome	*P* Value	Major Allele	Minor Allele	Minor Allele Freq.
EWS	A05:89580589	A05	2.67 × 10^−6^	C	A	0.10
EWS	A05:89717711	A05	5.28 × 10^−7^	T	C	0.19
EWS	A05:89762730	A05	5.14 × 10^−7^	C	G	0.20
EWS	A05:89762936	A05	8.70 × 10^−7^	G	A	0.19
EWS	A05:89984532	A05	1.62 × 10^−6^	A	G	0.19
EWS	A05:90061632	A05	7.20 × 10^−6^	G	A	0.17
EWS	A05:90150044	A05	1.05 × 10^−6^	A	G	0.21
EWS	A11:86279835	A11	7.42 × 10^−7^	A	G	0.11
CWS	A01:92213571	A01	2.31 × 10^−6^	A	G	0.18
CWS	A05:4580354	A05	2.80 × 10^−6^	T	A	0.42
CWS	A11:45127798	A11	1.46 × 10^−6^	T	C	0.06
LT	D03:20786242	D03	2.65 × 10^−6^	T	C	0.24
LT	D03:25327187	D03	1.71 × 10^−6^	A	T	0.18
LT	D03:25327414	D03	1.45 × 10^−6^	T	C	0.18
LT	D03:25669014	D03	2.66 × 10^−6^	A	C	0.22
LT	D03:25729410	D03	3.96 × 10^−7^	A	G	0.21
LT	D03:25991794	D03	4.04 × 10^−7^	A	G	0.19

## Supplementary Materials

Supplementary materials can be found at https://www.mdpi.com/1422-0067/20/16/3888/s1.

## Abbreviations

GWAS	Genome-wide association study
GLM	General linear model
FASTLMM	Factored spectrally transformed linear mixed model
EWS	Euphylla wilting score
CWS	Cotyledon wilting score
LT	Leaf temperature
LC	Leaf chlorophyll content
YZR	the Yangtze River basin region
YR	the Yellow River basin region
NEM	northern specific early maturation region
NW	northwestern inland region
FR	Foreign region
PCA	Principal component analysis
LD	Linkage disequilibrium
FH	Favorable haplotype
UFH	Unfavorable haplotype
HH	Heterozygous haplotype
MAF	Minor allele frequency
RWC	Relative water content
FDR	False discovery rate
DEG	Differential expression analysis
