# Supplementary material for: Analysis of Drought Tolerance and Associated Traits in Upland Cotton at the Seedling Stage
Source: Int J Mol Sci. 2019 Aug 9;20(16):3888. doi: 10.3390/ijms20163888 (PMC6720584; doi:10.3390/ijms20163888)
Supplement: Supplementary file 1 [file ijms-20-03888-s001.zip › Supplementary files/Supplementary Figure S1.pdf]

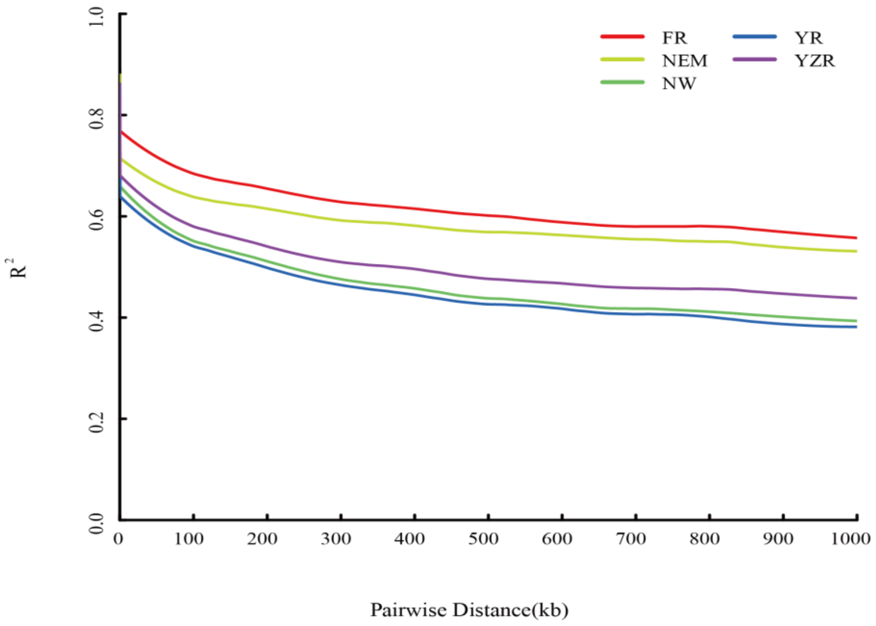

Figure S1. The mean LD decay rate was estimated as the squared correlation coefficient (R^2) using all pairs of SNPs located within approximately 400 kb of physical distance in genomic regions in five subpopulations of 316 upland cotton accessions
